# Molecular characterization of African swine fever virus from domestic pigs in northern Tanzania during an outbreak in 2013

**DOI:** 10.1007/s11250-014-0628-z

**Published:** 2014-07-05

**Authors:** Gerald Misinzo, David E. Kwavi, Christopher D. Sikombe, Mariam Makange, Emma Peter, Amandus P. Muhairwa, Michael J. Madege

**Affiliations:** 1 Department of Veterinary Microbiology and Parasitology, Faculty of Veterinary Medicine, Sokoine University of Agriculture, P.O. Box 3019, Morogoro, Tanzania; 2 Department of Veterinary Medicine and Public Health, Faculty of Veterinary Medicine, Sokoine University of Agriculture, Morogoro, Tanzania; 3 Directorate of Veterinary Services, Ministry of Livestock and Fisheries Development, Zonal Veterinary Centre, Arusha, Tanzania

**Keywords:** African swine fever, African swine fever virus, Asfarviridae, Tanzania, Domestic pig, Phylogeny

## Abstract

African swine fever (ASF) is an acute, highly contagious and deadly viral hemorrhagic fever of domestic pigs caused by African swine fever virus (ASFV), a double-stranded DNA virus of the family *Asfarviridae*. In this study, molecular diagnosis and characterization of outbreak ASFV in northern Tanzania, was performed on spleen, lymph node, kidney, and heart samples collected in June and July 2013 from domestic pigs that died during a hemorrhagic disease outbreak. Confirmatory diagnosis of ASF was performed using polymerase chain reaction (PCR) by partial amplification of *B646L* gene of ASFV encoding the major capsid protein p72 using PPA1/PPA2 primers. PCR using PPA1/PPA2 primers produced an expected PCR product size, confirming ASF outbreak in northern Tanzania. In addition, nucleotide amplification and sequencing, and phylogenetic reconstruction of the variable 3′-end of the *B646L* gene and complete *E183L* gene encoding the inner envelope transmembrane protein p54 showed that the 2013 outbreak ASFV from northern Tanzania were 100 % identical and clustered into ASFV *B646L* (p72) and *E183L* (p54) genotype X. Furthermore, the tetrameric amino acid repeats within the central variable region (CVR) of the *B602L* gene coding for the J9L protein had the signature BNBA(BN)_5_NA with a single novel tetramer NVDI (repeat code N). The results of the present study confirm an ASF outbreak in northern Tanzania in the year 2013 and show that the present outbreak ASFV is closely related to other ASFV from ticks, warthogs, and domestic pigs previously reported from Tanzania.

## Introduction

African swine fever (ASF) is an acute, highly contagious and deadly viral hemorrhagic fever of domestic pigs caused by African swine fever virus (ASFV) (Costard et al. 2013). ASFV is an enveloped double stranded DNA virus classified into the family *Asfarviridae* (Dixon et al. 2005). Depending on the ASFV strain, ASF morbidities and mortalities can reach 100 %, making ASF the most serious constraint for domestic pig production, food security, and livelihood (Penrith 2009). Although ASF was first described in Africa and is still endemic in many African countries, devastating transcontinental spread to Asian, European, and South American countries has occurred (Costard et al. 2013; Rowlands et al. 2008).

In eastern and southern African countries, ASF is naturally maintained in a sylvatic cycle involving warthog *Phacochoerus africanus* and the soft argasid tick *Ornithodoros moubata*, and transmission to domestic pigs leading to outbreaks is either through infected tick bites, feeding contaminated warthog carcasses or contact with warthog feces (Costard et al. 2009, 2013). Because of the involvement of this sylvatic cycle in the emergence of ASF, most outbreaks have been reported to start in the vicinity of National Parks (Penrith et al. 2007; Costard et al. 2013; Okoth et al. 2013). Transboundary spread of ASF during outbreaks within eastern Africa is mainly attributed to horizontal transmission between pigs due to uncontrolled pig and pig products movements, swill feeding, and lack of biosecurity measures (Wambura et al. 2006; Misinzo et al. 2011, 2012b).

After the isolation of ASFV in *O. moubata* ticks in the Serengeti National Park by Plowright et al. (1969), sporadic ASF outbreaks have been reported to occur at irregular intervals spanning several years (Wilson and Swai 2013). Since 2000, the frequency of reported ASF outbreaks in Tanzania has increased. Sporadic ASF outbreaks were reported in different regions including Dar es Salaam and Mbeya in 2001, Arusha in 2003, Kigoma in 2004, Mwanza in 2005 and Morogoro, Coast, and Dar es Salaam in 2008 and Arusha in 2009 (Wambura et al. 2006; Misinzo et al. 2011, 2012a, b). In 2010, an ASF outbreak was reported in Kyela of the Southern highland zone region of Mbeya that later on spread to other Southern highland zone regions of Rukwa and Iringa and Morogoro and Dar es Salaam regions of eastern Tanzania (Misinzo et al. 2012b). The 2010 ASF outbreak persisted until 2013 decimating pig stocks in the Southern highland zone, indicating the shift from sporadic to endemic occurrence of ASF in Tanzania (Sikombe 2013). The presence of asymptomatic pigs infected by virulent ASFV, possibly due to breed-related resistance (Uttenthal et al. 2013), may be one of the factors that contribute to the observed persistence of ASF in the Southern highland zone. The course of ASF may vary from acute, subacute, or chronic forms depending on the virulence of ASFV, host factors, and immunological status of pigs (Gómez-Villamandos et al. 2013). All ASF outbreaks in Tanzania reported between 2000 and 2013 are related to highly virulent ASFV strains that cause the acute form of ASF accompanied with high mortality rates (Wambura et al. 2006; Misinzo et al. 2011, 2012b).

Several virological tests are currently available for the diagnosis of ASF by detecting live ASFV, antigen and genome, including virus isolation, enzyme-linked immunosorbent assay (ELISA), fluorescent antibody assays, polymerase chain reaction (PCR), and isothermal amplification assays (Oura et al. 2013). Molecular differentiation between ASFV primarily relies on PCR amplification and nucleotide sequencing of the variable 3′-end of the *B646L* gene encoding the major capsid protein p72 (Bastos et al. 2003; Boshoff et al. 2007; Lubisi et al. 2007). Twenty two different genotypes (I to XXII) of ASFV have been identified based on nucleotide sequencing of the *B646L* (p72) gene (Bastos et al. 2003; Boshoff et al. 2007). The 22 ASFV the *B646L* (p72) genotypes can also be distinguished using microarray (Leblanc et al. 2013). Further subtyping of ASFV *B646L* (p72) genotypes into subgroups is achieved by nucleotide sequencing of the complete *E183L* gene encoding the inner envelope transmembrane protein p54 and analysis of the tetramer amino acid repeats within the hypervariable central variable region (CVR) of the *B602L* gene coding for the J9L protein (Irusta et al. 1996; Phologane et al. 2005; Nix et al. 2006; Lubisi et al. 2007; Gallardo et al. 2009). Genotype I ASFV is confined to Europe, South America, the Caribbean, and West Africa whereas viruses belonging to all the 22 known ASFV genotypes have been restricted to southern and eastern Africa (Costard et al. 2013). However, genotype IX ASFV has been described in western Africa (Gallardo et al. 2011a) and genotype II ASFV have spread to Madagascar, the Caucasus region, and Russian Federation Costard et al. 2013). In East and Southern Africa, some ASFV genotypes are country-specific, while others have transboundary distributions (Costard et al. 2009). Tanzanian outbreak ASFV belong into *B646L* (p72) genotypes II, IX, X, XV, and XVI (Lubisi et al. 2005; Misinzo et al. 2011, 2012a, b; Uttenthal et al. 2013).

In the present study, we report a 2013 deadly outbreak of ASF in domestic pigs in northern regions of Arusha and Kilimanjaro, Tanzania. Furthermore, we report the diagnosis and molecular characterization of 2013 outbreak ASFV in northern Tanzania based on partial amplification of the p72 gene using PCR and nucleotide sequencing of the variable 3′-end of the *B646L* gene, the complete *E183L* gene, and analysis of the tetramer amino acid repeats within the CVR. Our results indicate that the 2013 northern Tanzania outbreak ASFV belongs to *B646L* (p72) and *E183L* (p54) genotype X, has a unique CVR signature BNBA(BN)_5_NA including a single novel tetramer NVDI (repeat code N) and is very closely related to other ASFV previously described from ticks, warthogs, and domestic pigs in Tanzania.

## Materials and methods

### Study area and sampling

**Fig. 1:**
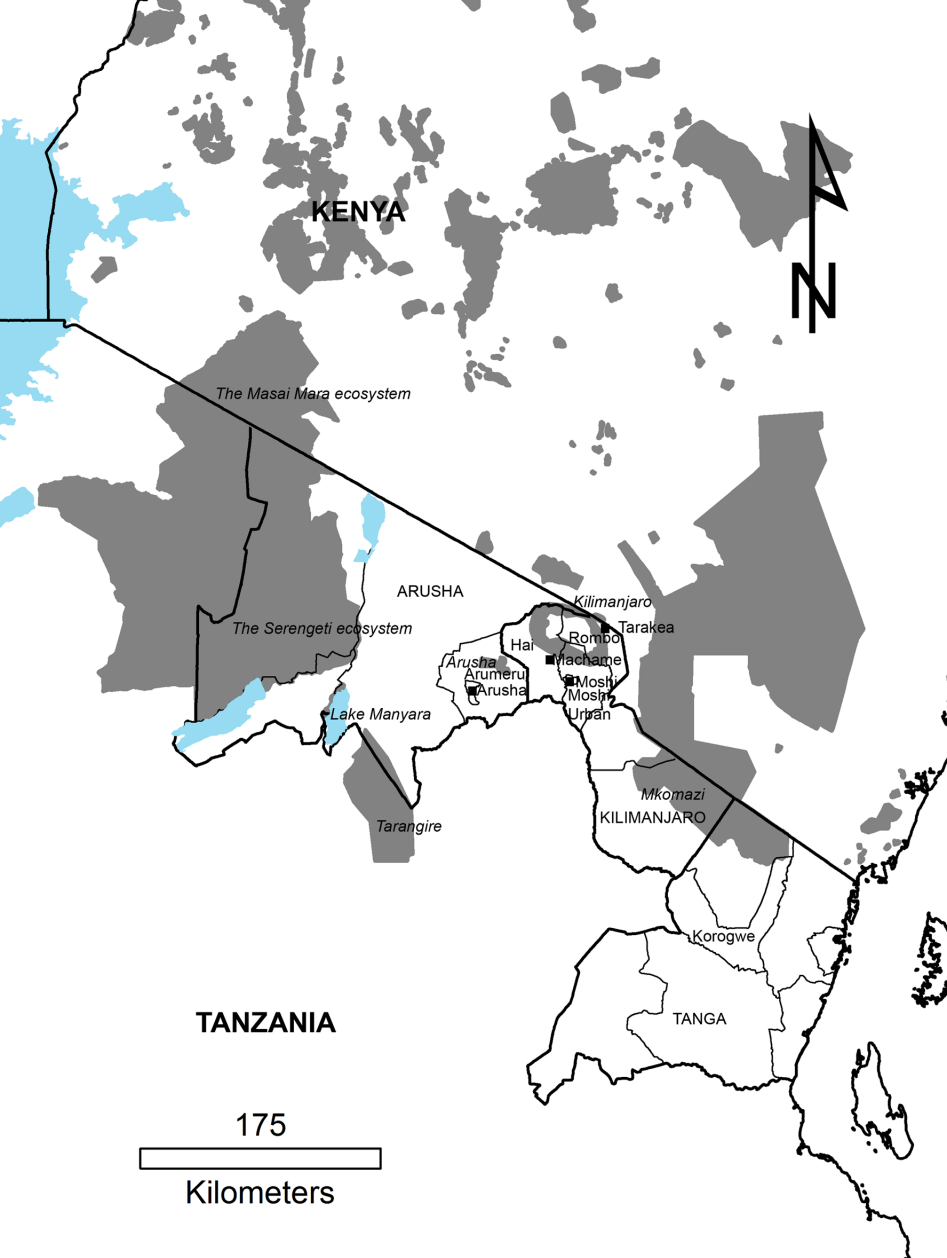
African swine fever (ASF) sampling sites in northern Tanzania. Tissue samples were obtained from dead domestic pigs during the 2013 ASF outbreak in northern Tanzanian regions of Kilimanjaro and Arusha. Samples were obtained from different locations including Arusha (in Arusha district), Machame (Hai), Tarakea (Rombo), and Moshi (Moshi Urban). The regions in northern Tanzania are indicated by *all caps* while National Parks are *shaded gray* and labeled in *italics*

This study was conducted in the northern Tanzania regions of Arusha and Kilimanjaro after a reported hemorrhagic disease outbreak in domestic pigs with clinical and gross morphological presentations suggestive of ASF. Spleen, lymph node, kidney, and heart were collected from dead pigs from different farms in Moshi city (the ASFV strain is identified as TAN/13/Moshi), Rombo town (TAN/13/Rombo), Nshara village of Machame ward in Hai district (TAN/13/Machame), and Arusha city (TAN/13/Arusha) during postmortem examinations (Fig. 1). Tissues were collected and placed in separate containers from a total of 13 pigs from Moshi city (*n* = 2), Rombo town (*n* = 4), Machame ward (*n* = 3), and Arusha city (*n* = 4). Samples were transported on ice and reached the laboratory in Morogoro within 24 h. Upon arrival, 1 g from each of the spleen, lymph node, kidney, and heart belonging to the same pig were pooled into sterile petri dishes and chopped using sterile scalpel blades after addition of 5:1 (*v*/*v*) sterile phosphate buffer saline (PBS) filtered through a 0.22-μm Minisart syringe filter (Sartorius Stedim Biotech, Goettingen, Germany). Afterwards, homogenized tissue samples were centrifuged at 6,000 *g* for 5 min and the supernatants aliquoted into cryovials before being stored at −80 °C.

### DNA extraction and amplification

Frozen aliquots of pooled spleen, lymph node, kidney, and heart tissue homogenates were thawed and DNA extraction performed using a QiaAmp nucleic extraction kits (Qiagen, Hilden, Germany), following the manufacturer’s instructions. During nucleic acid extraction and PCR, the homogenized tissue suspension containing the TAN/12/Ifakara (Misinzo et al. 2012b) was used as positive control and PBS as negative control. PCR detection of ASFV was performed using primers PPA1 and PPA2 that partially amplify the *B646L* gene encoding the major capsid protein p72, as previously described by Aguero et al. (2003). The expected size of the PCR product using primers PPA1 and PPA2 primers is 257 bp. PCR for the molecular characterization of ASFV was performed to amplify the (i) variable 3′-end of the *B646L* gene encoding the major capsid protein p72 using primers p72U and p72D, (ii) complete *E183L* gene encoding the inner envelope transmembrane protein p54 using primers PPA89 and PPA722, and (iii) the tetramer amino acid repeats within the hypervariable CVR of the *B602L* gene using ORF9L-F and ORF9L-R primers, as previously described by Gallardo et al. (2009). All PCR amplifications were performed using AccuPower PCR premix (Bioneer, Daejeon, Republic of Korea) on a GeneAmp PCR systems 9700 (Applied Biosystems, Foster City, CA). Afterwards, PCR products were electrophoresed in a 2 % agarose gel mixed with GelRed nucleic acid stain (Phenix Research Products, Candler, NC) before visualization and imaging using a BioDoc-It imaging system (UVP, Upland, CA).

### Nucleotide sequencing

PCR products from *B646L* (p72), *E183L* (p54), and CVR were purified from agarose gels using a NucleoSpin gel and PCR clean-up kit (Macherey-Nagel, Düren, Germany) and subjected to dideoxynucleotide cycle sequencing by using Big Dye Terminator Cycle Sequencing Kit Version 3.1 (Applied Biosystems, Foster City, CA). Products from dideoxynucleotide cycle sequencing reaction were purified by ethanol precipitation and separated on a 3500 Genetic Analyzer (Applied Biosystems, Foster City, CA). Chromatograms for both the forward and the reverse primer reactions were read using Sequence Scanner v1.0 software (Applied Biosystems, Foster City, CA). The sequence from the forward primer and the reverse complement sequence of the reverse primer were manually overlapped using a text editor to obtain a single consensus sequence delimited by the forward and reverse primers.

### Molecular characterization of ASFV using the *B646L* (p72), *E183L* (p54), and CVR approach

The nucleotide sequence of *B646L* (p72), *E183L* (p54), and CVR from the 2013 ASF outbreak in northern Tanzania were submitted to GenBank and given accession numbers. The similarity search of the obtained nucleotide sequences against other ASFV sequences at GenBank database was performed using BLASTn (version 2.2.29). BLASTn compares nucleotide sequences to sequence databases and calculates the statistical significance of matches. The nucleotide sequences of the *B646L* (p72), *E183L* (p54), and CVR genes of northern Tanzanian 2013 outbreak ASFV were aligned with other Tanzanian ASFV nucleotide sequences available at GenBank (Table 1) using ClustalW algorithm in BioEdit (Ibis Biosciences, Carlsbad, CA). The clustering pattern of ASFV was determined by neighbor-joining method using the Kimura-2-parameter option implemented within MEGA 5 (Tamura et al. 2011). Phylogeny was inferred following 1,000 bootstrap replications. The CVR nucleotide sequences of the northern Tanzanian 2013 outbreak ASFV were translated and coded to obtain signatures based on previously reported codes (Nix et al. 2006; Boshoff et al. 2007; Misinzo et al. 2011). A similarity search against other ASFV amino acid sequences was performed using BLASTp (v2.2.2.29).

**Table 1:** Tanzanian African swine fever virus isolates used for the construction of phylogenetic trees based on partial *B646L* (p72) gene sequences

Isolate	Host species	Year of isolation	Town	p72 gene Genbank accession number	p72 genotype	Reference
TAN/10/Kyela	Pig	2010	Kyela	JX391987	II	Misinzo et al. 2012b
TAN/11/Ludewa	Pig	2011	Ludewa	JX391990	II	Misinzo et al. 2012b
TAN/12/Ifakara	Pig	2012	Ifakara	JX391992	II	Misinzo et al. 2012b
TAN/13/Iringa	Pig	2013	Iringa	KF834193	II	Sikombe 2013
TAN 2005.1	Pig	2005	Mwanza	JX403640	IX	Unpublished
KIRT 89/4	Tick	1989	Kirawira	AY351513	X	Lubisi et al. 2005
KIRW 89/1	Warthog	1989	Kirawira	AY351514	X	Lubisi et al. 2005
TAN/Kwh12	Warthog	1968	Kirawira	AF301546	X	Bastos et al. 2003
TAN 2004.1	Pig	2004	Kigoma	JX403648	X	Unpublished
TAN/09/Longido	Pig	2009	Longido	JX262383	X	Misinzo et al. 2012a
TAN/13/Moshi	Pig	2013	Moshi	KF706360	X	This study
TAN/13/Rombo	Pig	2013	Rombo	KF706361	X	This study
TAN/13/Machame	Pig	2013	Machame	KF706362	X	This study
TAN/13/Arusha	Pig	2013	Arusha	KF706363	X	This study
Tan/1/01	Pig	2001	Dar es Salaam	AY494552	XV	Lubisi et al. 2005
TAN/08/Mazimbu	Pig	2008	Mazimbu	GQ410765	XV	Misinzo et al. 2011
TAN/2003/1	Pig	2003	Arusha	AY494550	XVI	Lubisi et al. 2005

## Results

### Epidemiology of ASF in Arusha and Kilimanjaro

ASF was first reported in Tarakea (Rombo district), located at the border between Tanzania and Kenya in May 2013 (Fig. 1). Afterwards, ASF spread from Tarakea to neighboring Useri and Mashati wards of Rombo district before it was reported in Arusha city on 06 June 2013 in villages surrounding the Arusha dampo, including Muriet and Engosengiu of Sokoni One ward. On 18 June 2013, ASF was reported in a number of districts including Moshi Rural, Moshi Urban (Mbokomu ward), Arusha, and Arumeru (Sekei and Olga Lai wards) districts (Fig. 1). Between July and August 2013, ASF had spread to Korogwe and Hai (Machame ward) districts. The number of recorded domestic pig death due to ASF is shown in Table 2. By September 2013, there were no reports on new cases. In Rombo and Arusha districts, ASF spread as a result of feeding pork leftovers from hotels, restaurants, and homes. The spread between the different districts is attributed to introductions of infected animals bought at a cheaper price from ASF areas under quarantine due to ineffective law enforcement.

**Table 2:** Mortalities resulting from an African swine fever outbreak in domestic pigs at different locations in Northern Tanzania between May and August 2013

Region	District	Deaths
Arusha	Arusha	945
	Arumeru	5
Kilimanjaro	Rombo	4462
	Moshi Rural	859
	Moshi Urban	457
	Hai	9
Tanga	Korogwe	58

### Clinical signs and postmortem findings in domestic pigs with ASF

**Fig. 2:**
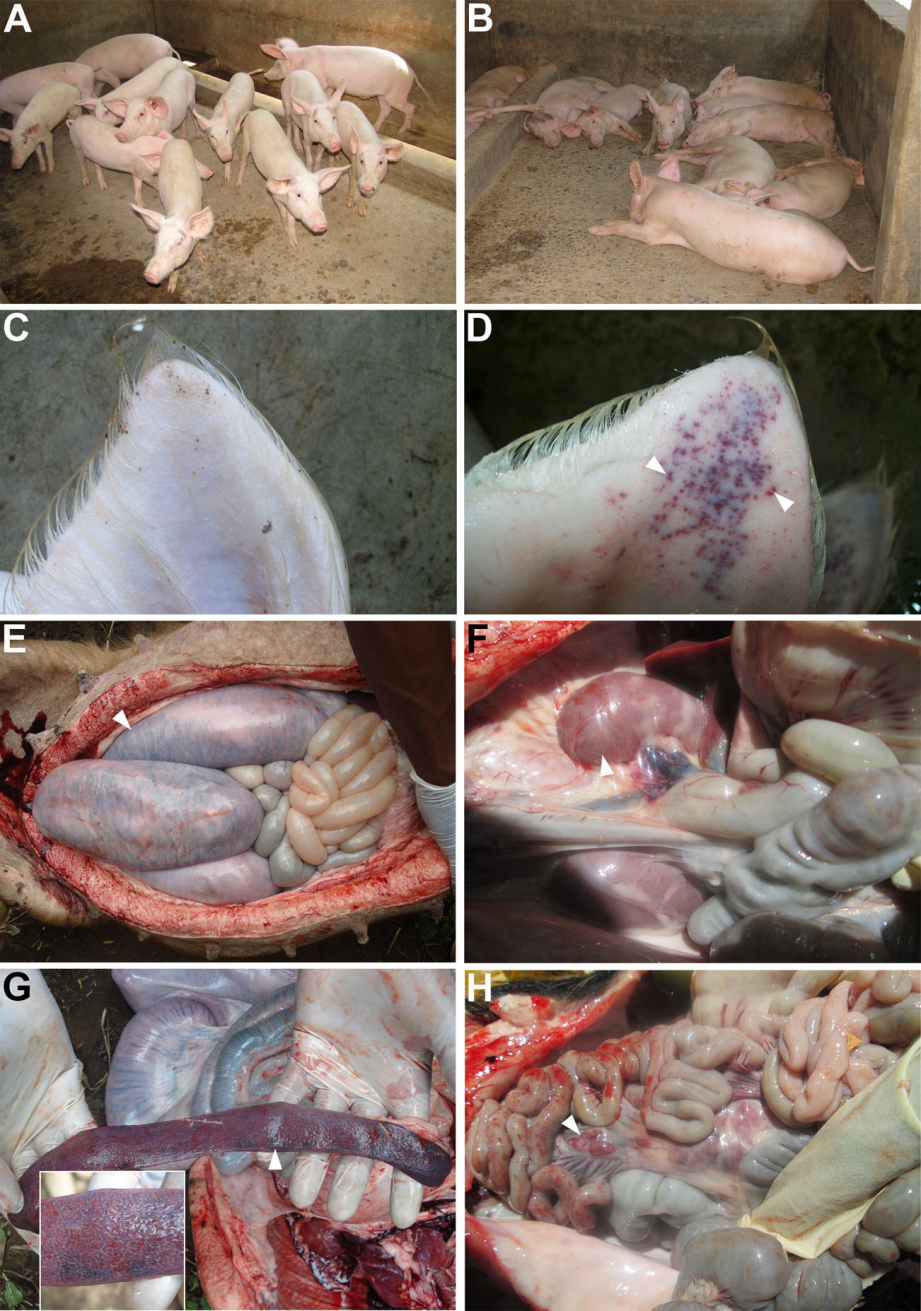
Clinical signs and postmortem findings in domestic pigs with African swine fever (ASF). Pigs in one of the piggery units where ASF had just started showing (**a**) alert pigs in unaffected pens and (**b**) recumbent pigs with ASF. An ear of a healthy pig is shown in (**c**) while an ear with marked cutaneous congestion (indicated with an *arrow head*) in pigs with ASF is shown in (**d**). In addition, pigs with ASF showed hemorrhages (indicated by an *arrow head*) of the intestines (**e**), kidneys (**f**), spleen (**g**), and mesenteric lymph nodes (**h**). **g** Enlargement of the spleen (splenomegaly) was observed in pigs with ASF

The main clinical signs presented by the sick domestic pigs in the visited farms included hind leg weakness, recumbence, dyspnea, anorexia, and erythema and cyanosis of the skin (Fig. 2). Domestic pigs of all ages were affected although severe clinical signs were observed in adults than piglets. Abortion was frequently observed in pregnant sows. At postmortem, straw-colored or blood-tinged fluid was observed in the pleura, pericardium, and peritoneal cavities. In addition, splenomegaly, petechiations of the heart and kidney, and hemorrhagic heart, kidneys, liver, intestines, and lymph nodes especially the mesenteric and the hepatogastric lymph nodes were observed (Fig. 2).

### Confirmatory diagnosis of ASFV using PCR

An ASF diagnostic PCR previously reported by Aguero et al. (2003) was used to confirm the presence of ASF in samples collected from dead pigs in Moshi, Rombo, Machame, and Arusha. With the exception of tissues from one pig in Arusha, all pooled tissues belonging to 12 pigs from Moshi city (*n* = 2), Rombo town (*n* = 4), Machame ward (*n* = 3), and Arusha city (*n* = 3) were positive. The PCR products of ASFV from Moshi, Rombo, Machame, and Arusha obtained after performing diagnostic PCR using PPA1/2 primers were 257 nucleotides long (Misinzo et al. 2014).

### Molecular characterization of ASF based on the *B646L* (p72), *E183L* (p54), and CVR approach

Nucleotide amplification and sequencing of the variable 3′-end of the *B646L* gene encoding the major capsid protein p72, complete *E183L* gene encoding the inner envelope transmembrane protein p54, and the hypervariable CVR of the *B602L* gene was performed on tissue samples collected from dead pigs in Moshi, Rombo, Machame, and Arusha. Twelve 2013 northern Tanzanian ASFV DNA sequences were deposited at GenBank including KF706356 (p54; TAN/13/Moshi), KF706357 (p54; TAN/13/Rombo), KF706358 (p54; TAN/13/Machame), KF706359 (p54; TAN/13/Arusha), KF706360 (p72; TAN/13/Moshi), KF706361 (p72; TAN/13/Rombo), KF706362 (p72; TAN/13/Machame), KF706363 (p72; TAN/13/Arusha), KF706364 (CVR, TAN/13/Moshi), KF706365 (CVR, TAN/13/Rombo), KF706366 (CVR, TAN/13/Machame), and KF706367 (CVR, TAN/13/Arusha).

**Fig. 3:**
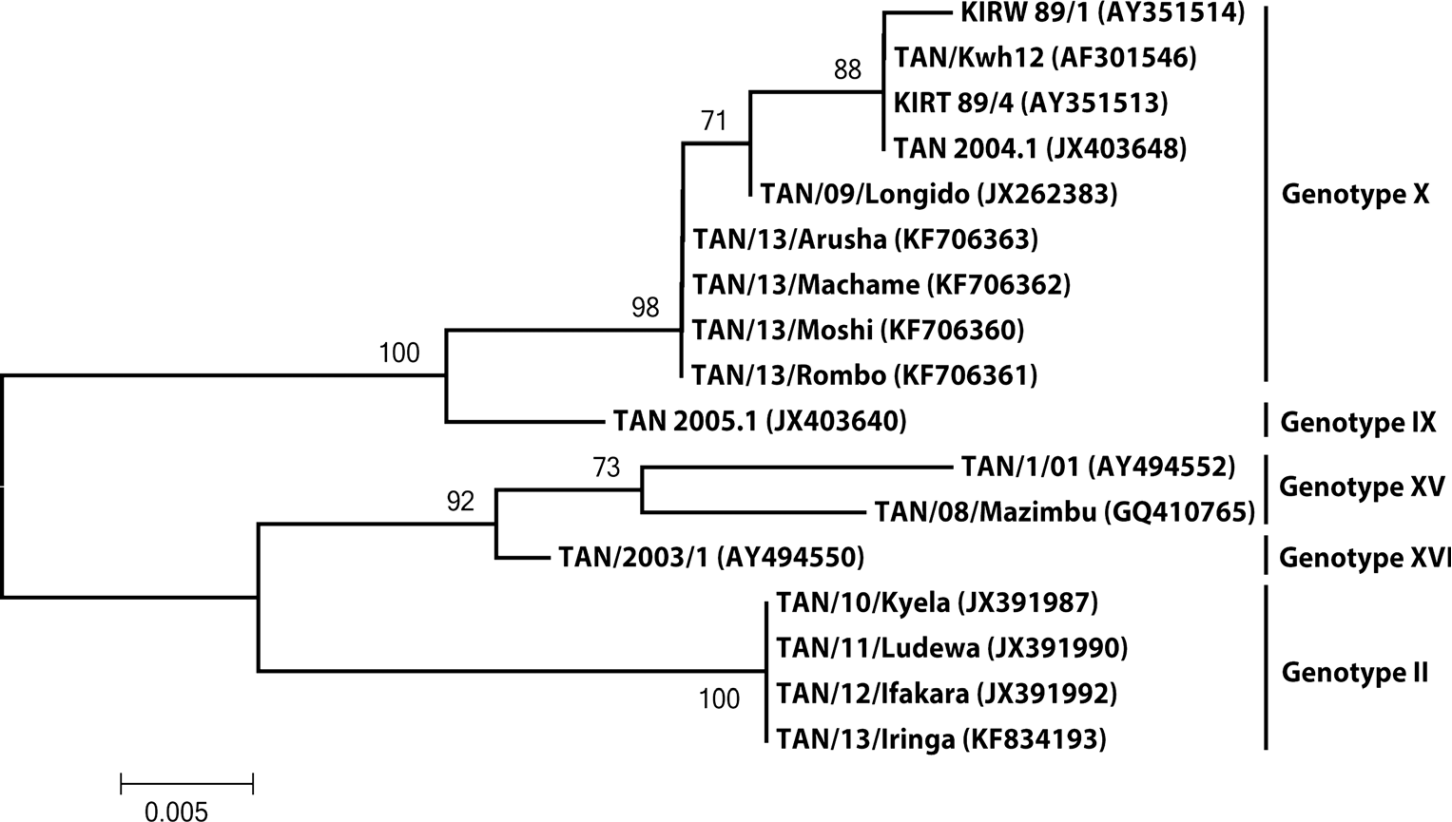
Neighbor-joining tree depicting partial *B646L* (*p72*) gene relationships of African swine fever viruses from Tanzania. Phylogeny was inferred following 1,000 bootstrap replications, and the node values show percentage bootstrap support. *Scale bar* indicates nucleotide substitutions per site. The Genbank accession numbers for the different *B646L* (*p72*) genes are indicated in *parenthesis*

ASFV from Arusha (TAN/13/Arusha) and Kilimanjaro (TAN/13/Moshi, TAN/13/Rombo and TAN/13/Machame) regions were 100 % identical in their *B646L* (p72), *E183L* (p54), and *B602L* (CVR) nucleotide sequences. The similarity search of the *B646L* (p72) nucleotide sequence obtained in this study against other ASFV sequences at GenBank using BLASTn showed 444 out of 445 nucleotide identity with TAN/09/Longido ASFV that caused an ASF outbreak in domestic pigs in northern Tanzania in 2009 (Misinzo et al. 2012a). When phylogenetic analysis of the Tanzanian ASFV *B646L* (p72) nucleotide sequences was performed together with the nucleotide sequences obtained from this study, the 2013 ASFV from Moshi, Rombo, Machame, and Arusha clustered together with ASFV belonging to genotype X (Fig. 3). The 2013 ASFV from Moshi, Rombo, Machame, and Arusha also clustered into genotype X when phylogenetic analysis was performed using the *E183L* (p54) nucleotide sequences (data not shown).

The CVR nucleotide sequences of the northern Tanzanian 2013 outbreak ASFV were translated and coded to obtain their signatures. ASFV tetrameric amino acid repeats within CVR that have previously been reported in ASFV include CAST/CASI/CVST/CTST (repeat code A), CADT/CTDT (B), GAST/GANT (C), CASM (D), CANT (F), CTNT (G), NEDT (M), NVDT/NVGT/NVNT (N), NANI/NADI/NASI (O), RAST (H), SAST (S), NVNT (T), NAST/NADT/NANT/NAVT (V), SADT/SVDT (W), NIDT/NTDT (U), and NTDI (X) (Nix et al. 2006; Boshoff et al. 2007; Misinzo et al. 2011). The CVR of 2013 Tanzanian strains TAN/13/Moshi, TAN/13/Rombo, TAN/13/Machame, and TAN/13/Arusha had five different tetrameric amino acid repeats, namely CAST (A), CADT/CTDT (B), and NVDT/NVDI (N) that were repeated 16 times with the signature BNBA(BN)_5_NA.

## Discussion

In this study, the molecular diagnosis and characterization of ASFV from domestic pigs that died of a hemorrhagic disease outbreak between May and August 2013 in northern Tanzania was performed. The results obtained from the present study confirm ASF outbreak in Arusha and Kilimanjaro regions located in the northern Tanzania. ASFV in domestic pigs was confirmed by nucleotide amplification, sequencing, and phylogenetic reconstruction of ASFV *B646L* (p72) and *E183L* (p54) genes, and translation of the *B602L* (CVR) gene. The obtained *B646L* (p72), *E183L* (p54), and *B602L* (CVR) nucleotide sequences of 2013 outbreak ASFV from northern Tanzania were 100 % identical and clustered into ASFV *B646L* (p72) and *E183L* (p54) genotype X. Furthermore, the tetrameric amino acid repeats within the CVR of the *B602L* gene had the signature BNBA(BN)_5_NA including a single novel tetramer NVDI (repeat code N).

The present ASF outbreak started in May 2013 in Kilimanjaro region before spreading to Arusha region in June 2013. By September 2013, no new cases of ASF were recorded in Kilimanjaro and Arusha. The sporadic nature and short longevity of the 2013 ASF outbreak is comparable to previous outbreaks of 2001, 2003, 2004, 2005, 2008, and 2009 that lasted for a few months before resolving (Wambura et al. 2006; Misinzo et al. 2011, 2012a). In Tanzania, the only recorded outbreak that persisted longer was the ASF outbreak between 2010 and 2013 that started in Mbeya region and later on spread to Iringa, Rukwa, Dar es Salaam, and Morogoro regions (Misinzo et al. 2012b; Sikombe 2013). The 2010 to 2013 ASF outbreak was caused by a *B646L* (p72) genotype II ASFV that had previously not been described in Tanzania and eastern Africa (Misinzo et al. 2012b; Sikombe 2013). The *B646L* (p72) and *E183L* (p54) genes of this Tanzanian genotype II ASFV are identical to the Georgia 2007/1 virus and clusters into this genotype with other ASFV from Mozambique, Madagascar, and Mauritius (Misinzo et al. 2012b; Uttenthal et al. 2013). Before the genotype II introduction, Tanzanian outbreak ASFV clustered into *B646L* (p72) genotypes IX in 2005, X in 2004 and 2009, XV in 2001 and 2008, and XVI in 2003 (Lubisi et al. 2005; Wambura et al. 2006; Misinzo et al. 2011, 2012a). The ASFV identified in this study clusters into *B646L* (p72) genotype X, similar to other viruses that have caused ASF outbreak in Tanzania in 2009 in Longido (northern Tanzania), 2004 in Kigoma (western Tanzania), and other ASFV recovered from ticks and warthogs in Serengeti National Park (northern Tanzania) (Lubisi et al. 2005; Wambura et al. 2006; Misinzo et al. 2012a). Genotype X is a sylvatic cycle-associated genotype that comprises ASFV recovered from domestic pigs, warthogs and ticks in Burundi, Tanzania, and Kenya (Bastos et al. 2003; Lubisi et al. 2005; Lubisi et al. 2007; Gallardo et al. 2011b).

The Tanzanian 2013 ASFV reported in the present study originated from Tarakea, a town bordering Kenya and very close to the Kilimanjaro National Park. In addition, the Tanzanian 2013 ASFV is closely identical to the 2009 outbreak ASFV (TAN/09/Longido) from another town located within northern Tanzania. Alignment of 444 nucleotide long sequence of the variable 3′-end of the *B646L* (p72) gene of Tanzanian 2013 with 2009 ASFV show only a single nucleotide substitution (A→T). The close nucleotide identity between 2013 and 2009 outbreak ASFV indicates the presence of closely related viruses in northern Tanzania. Northern Tanzania has several wildlife protected areas (Fig. 1). ASFV diversity is generated during the sylvatic cycle of the virus (Jori et al. 2013). There is a possibility that viruses evolving in these wildlife protected areas spill over to the domestic pig population causing outbreaks such as the 2009 and 2013 outbreaks. It will be important to sequence the *E183L* (p54) and *B602L* (CVR) genes of TAN/09/Longido ASFV in order to understand the similarity between this virus and the 2013 viruses at these genomic locations. In addition, it will be important to perform the surveillance and genetic characterization of ASFV from warthogs and ticks found in Tanzanian National Parks in order to understand the relationship between sylvatic and outbreak ASFV.

To date, the CVR region has only been analyzed for the 2008 Tanzanian outbreak ASFV (Misinzo et al. 2011). The tetrameric amino acid repeats within the CVR of the *B602L* gene of the 2013 Tanzanian ASFV reported in this study had the signature BNBA(BN)_5_NA, which is distinct from the 2008 Tanzanian ASFV that had the signature AVUAVUVAVVUAVUVAVUVAVVUAVVUUUXV (Misinzo et al. 2011). BLASTp (v2.2.29) analysis using the deduced amino acid sequence showed 75 % overall amino acid identity with the CVR of Mozambican viruses SPEC/265 and Moz 94/1, that belong to genotype VI (Bastos et al. 2004; Nix et al. 2006). The CVR signature for SPEC/265 is AABABNABABNBABMA while that of Moz94/1 is AAAABABNABABNBTBA, indicating that the 2013 Tanzanian ASFV differ from Mozambican viruses.

Over the past two decades, the frequency of ASF outbreaks in Tanzania has increased. When the ASF outbreak in northern Tanzania (Kilimanjaro and Arusha) was reported, there was an ongoing outbreak of ASF in southern Tanzania (Mbeya, Iringa and Rukwa) (Misinzo et al. 2012b). Northern and southern Tanzania are the major pig-producing zones in Tanzania with highest pig population in Mbeya followed with Iringa, Ruvuma, and Kilimanjaro region (URT 2012). Pig husbandry has recently increased, for instance in Rombo district (Kilimanjaro region), pig farming has expanded as source of income to many households replacing coffee farming. The ASF outbreaks in southern and northern Tanzania has decimated the pig population affecting the food security and livelihoods of poor pig farmers.

It can be concluded from the results obtained in this study that the ASFV that caused the 2013 outbreak in Arusha and Kilimanjaro regions in northern Tanzania are related to other previously reported *B646L* (p72) genotype X eastern African sylvatic cycle viruses and differ from the genotype II ASFV reported in southern Tanzania. Furthermore, unrestricted pig movements and swill feeding continues to contribute to the spread of ASF outbreaks within Tanzania. The impact of ASF on the livelihood of farmers resulting from higher frequency and increased durations of ASF outbreaks in Tanzania is worth investigating.
